# Supplementary material for: Modified secreted alkaline phosphatase as an improved reporter protein for N-glycosylation analysis
Source: PLoS One. 2021 May 25;16(5):e0251805. doi: 10.1371/journal.pone.0251805 (PMC8148361; doi:10.1371/journal.pone.0251805)
Supplement: S1 Fig — (PDF) [file pone.0251805.s001.pdf]

Schematic description of modifications introduced to Secretable Alkaline Phosphatase (SEAP)

↓

MILGPCMLLLLLLLGLRLQLSLG IIPVEEENPDFWNREAAEALGAAKKLQPAQTAAKNLII
FLGDGMGVSTVTAARILKGQKKDKLGPEIPLAMDRFPYVALSKTYNVDKHVPDSGATATAYL
CGVKGNFQTIGLSAAARFNQCNTTRGNEVISVMNRAKKAGKSVGVVTTTRVQHASPAGTYAH
TVNRNWYSDADVPASARQEGCQDIATQLISNMDIDVILGGGRKYMFRMGTPDPEYPDDYSQG
GTRLDGKNLVQEWLAKRQGARYVWNRTELMQASLDPSVTHLMGLFEPGDMKYEIHRDSTLDP
SLMEMTEAALRLLSRNPRGFFLFVEGGRIDHGHHESRAYRALTETIMFDDAIERAGQLTSEE
DTLSLVTADHSHVFSFGGYPLRGSSIFGLAPGKARDRKAYTVLLYGNGPGYVLKDGARPDVT
ESESGSPEYRQQSAVPLDEETHAGEDVAVFARGPQAHLVHGVQEQTFIAHVMAFAACLEPYT
ACDLAPPAGTTDAAHPGRSRSKRLD - fusion peptide

Fusion peptide attached at C-terminus:

6xHis:

HHHHHH

HA:

YPYDVPDYA

GST:

MSPILGYWKIKGLVQPTRLLLEYLEEKYEEHLYERDEGDKWRNKKFELGLEFPNLPYYIDGD
VKLTQSMAIIRYIADKHNMLGGCPKERAEISMLEGAVLDIRYGVSRIAYSKDFETLKVDFLS
KLPEMLKMFEDRLCHKTYLNGDHVTHPDFMLYDALDVVLYMDPMCLDAFPKLVCFKKRIEAI
PQIDKYLKSSKYIAWPLQGWQATFGGGDHPPKSDLVPR

Native sequons are marked with GREEN colour (from which only the 2nd N-glycosylation site, at pos. 272, is occupied)

New sequons introduced by site-directed mutagenesis are marked with YELLOW colour

Changed amino acid residues are marked in sequons as RED letters

The arrow ↓ shows the signal peptide **MILGPCMLLLLLLLGLRLQLSLG** cleavage site
