# Supplementary material for: Modified secreted alkaline phosphatase as an improved reporter protein for N-glycosylation analysis
Source: PLoS One. 2021 May 25;16(5):e0251805. doi: 10.1371/journal.pone.0251805 (PMC8148361; doi:10.1371/journal.pone.0251805)
Supplement: S2 Fig — (PDF) [file pone.0251805.s002.pdf]

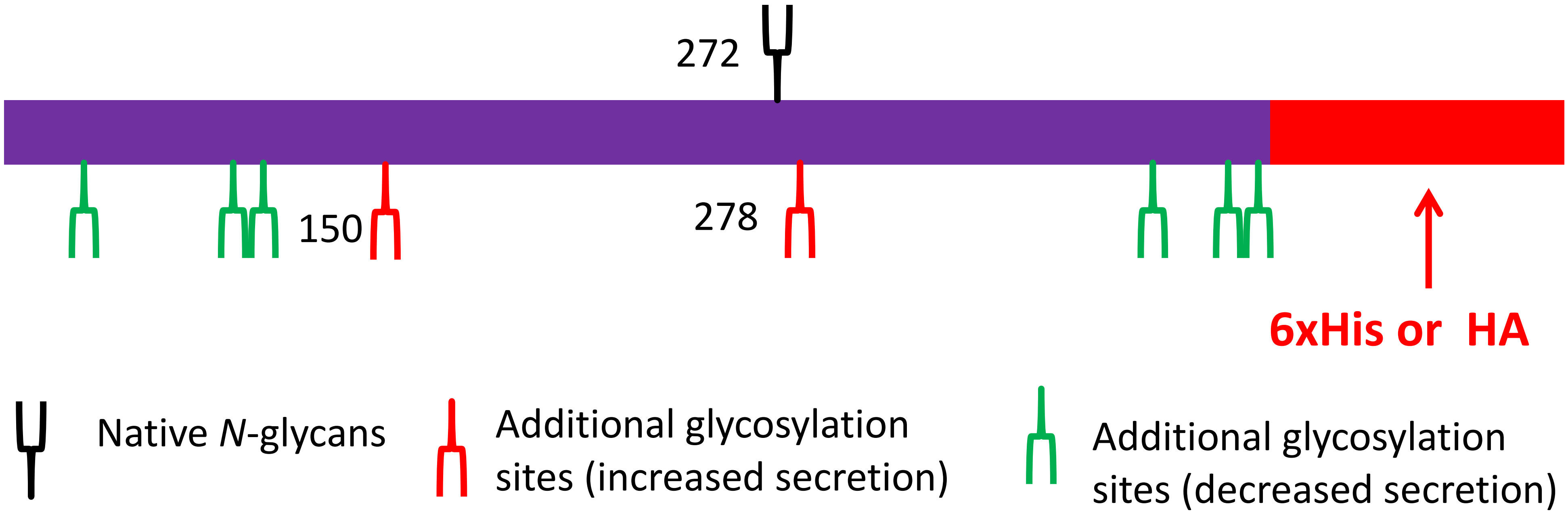

272
150
278
6xHis or HA
Native N-glycans
Additional glycosylation sites (increased secretion)
Additional glycosylation sites (decreased secretion)
