# Supplementary material for: Modified secreted alkaline phosphatase as an improved reporter protein for N-glycosylation analysis
Source: PLoS One. 2021 May 25;16(5):e0251805. doi: 10.1371/journal.pone.0251805 (PMC8148361; doi:10.1371/journal.pone.0251805)
Supplement: S1 Table — (PDF) [file pone.0251805.s003.pdf]

Primers used in this study

Primer	Sequence	Used for
SEAP-38-F	CTGGAACCGCacGGCAGCCGAG	introducing ^{38}Glu > ^{38}Thr point mutation
SEAP-38-R	AAGTCCGGGTTCTCCTCC	
SEAP-101-F	CCCATATGTGaatCTGTCCAAGACATACAATG	introducing ^{101}Ala > ^{101}Asn point mutation
SEAP-101-R	AAGCGGTCCATAGCCAGG	
SEAP-109-F	ATACAATGTAacCAAACATGTGCCAGACAG	introducing ^{109}Asp > ^{109}Thr point mutation
SEAP-109-R	GTCTTGGACAGAGCCACA	
SEAP-152-F	CGGCAACGAGacCATCTCCGTG	introducing ^{152}Val > ^{152}Ser point mutation
SEAP-152-R	CGTGTCGTGTTGCACTGG	
SEAP-278-F	TGAGCTCATGaacGCTTCCCTGG	introducing ^{278}Gln > ^{278}Asn point mutation
SEAP-278-R	GTGCGGTTCCACACATAC	
SEAP-477-F	CGGCGTGCAGaacCAGACCTTCATAGC	introducing ^{493}Pro > ^{493}Asn point mutation
SEAP-477-R	TGAACCAGGTGCGCCTGC	
SEAP-493-F	CTGCCTGGAGaaCTACACCGCCTGC	introducing ^{493}Pro > ^{493}Asn point mutation
SEAP-493-R	GCGGCGAAGGCCATGACG	
GST_Bgl-F	ctag***agatct*****ATG**TCCCCTATACTAGGTTATTGG	amplify GST insert for psiTest plasmid
GST_Nhe_STOP-R	ctag***gctagc*****TCA**acgcggaaccagatccg	
SEAP_Age_F	gatc***accggt***CACCATGATTC	amplify of HA and 6His SEAP inserts
SEAP_Bgl_6His_R	gcat***atgcat*****TCAATGGTGATGGTGATGGTG**ATCCAGACGCTTGGACCG	amplify 6His insert
SEAP_Nhe_HA_R	ctag***gctagc***TTA**TGCGTAGTCTGGTACGTCGTATGGGTA**atccagacgcttggaccg	amplify HA insert
