# Supplementary material for: Modified secreted alkaline phosphatase as an improved reporter protein for N-glycosylation analysis
Source: PLoS One. 2021 May 25;16(5):e0251805. doi: 10.1371/journal.pone.0251805 (PMC8148361; doi:10.1371/journal.pone.0251805)

Page #1. Results (all lines) are presented in Figure 1.

12 µg of 6His-SEAP (sample 1-3) eluted from Ni-NTA magnetic beads with PAGE sample buffer(1st line), 2xGlycobuffer(line 2) and imidazole (lane 3), respectively (all 3 samples are on the left side of Mw marker). In the middle (lane 4) - Mw markers, 200 kDa, 150 kDa, 100 kDa, 70 kDa, 50 KDa, 40 kDa, 25 kDa, 15 kDa. On the right side: control line 5 (anti-HA magnetic beads incubated in PAGE sample buffer), lane 6 - 3 µg of SEAP-HA eluted with PAGE sample buffer from HA-magnetic beads, lane 7 - 3 µg of SEAP-HA eluted with 2 x Glycobuffer from HA-magnetic beads.

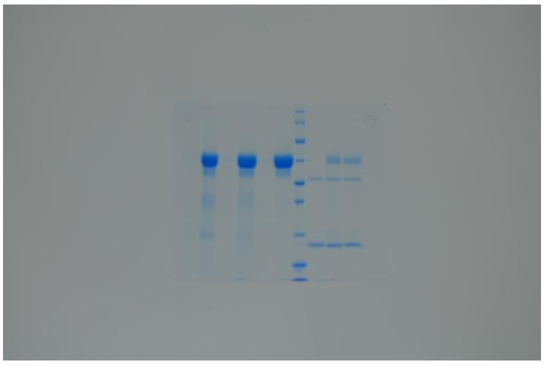

Page 2 used as an insert in Figure 2.

Purified variants of SEAP-6His (1µg of each) was applied to the gel. From lesf to right: line 1-SEAP-6His, lane 2-SEAP-6His with additional glysocylation site ate position 278, lane 3-SEAP-6His with additional glycosylation sites at positions 150 and 278. Mw markers in lane 4 (145 kDa, 116 kDa, 66.2 kDa, 45 kDa)

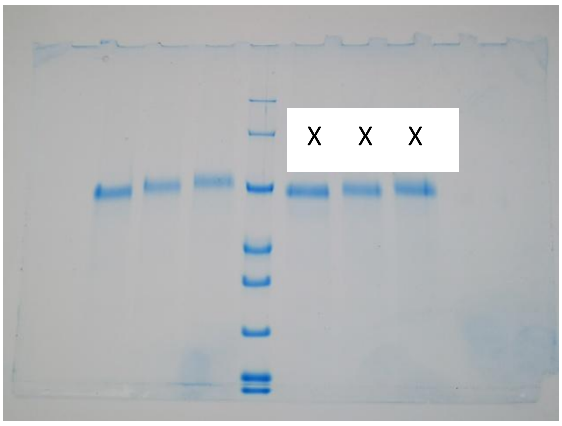

Page 3 used in Figure 3.

lane 4 - Mw markers, 200 kDa, 150 kDa, 100 kDa, 70 kDa, 50 KDa, 40 kDa, 25 kDa, 15 kDa. Lines 5,6 & 7 show SEAP-6His, SEAP-6-His_278 and SEAP-6His_150&278 variants, respectively. 6 µg was loaded on each line. These proteins were eluted from magnetic Ni-NTA beads with imidazole and used for enzymatic analysis.

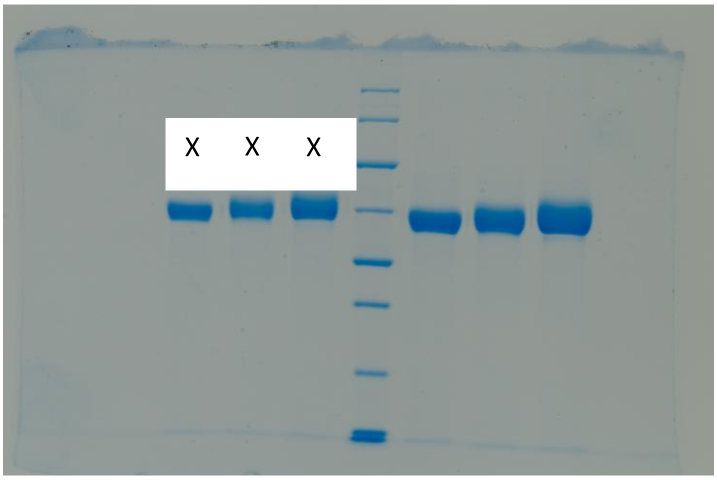

Supplement: S1 Raw images — (PDF) [file pone.0251805.s004.pdf]
