# Supplementary material for: Modified secreted alkaline phosphatase as an improved reporter protein for N-glycosylation analysis
Source: PLoS One. 2021 May 25;16(5):e0251805. doi: 10.1371/journal.pone.0251805 (PMC8148361; doi:10.1371/journal.pone.0251805)
Supplement: Attachment — Submitted filename: PlosOne 2021 responces-final.docx [file pone.0251805.s005.docx]

Journal Requirements:

When submitting your revision, we need you to address these additional requirements.

 1. Please ensure that your manuscript meets PLOS ONE's style requirements, including those for file naming. The PLOS ONE style templates can be found at

https://journals.plos.org/plosone/s/file?id=wjVg/PLOSOne_formatting_sample_main_body.pdf and

https://journals.plos.org/plosone/s/file?id=ba62/PLOSOne_formatting_sample_title_authors_affiliations.pdf

 The manuscript was re-formatted according to PLOS ONE requirements

2. PLOS ONE now requires that authors provide the original uncropped and unadjusted images underlying all blot or gel results reported in a submission’s figures or Supporting Information files. This policy and the journal’s other requirements for blot/gel reporting and figure preparation are described in detail at https://journals.plos.org/plosone/s/figures#loc-blot-and-gel-reporting-requirements and https://journals.plos.org/plosone/s/figures#loc-preparing-figures-from-image-files. When you submit your revised manuscript, please ensure that your figures adhere fully to these guidelines and provide the original underlying images for all blot or gel data reported in your submission. See the following link for instructions on providing the original image data: https://journals.plos.org/plosone/s/figures#loc-original-images-for-blots-and-gels.

In your cover letter, please note whether your blot/gel image data are in Supporting Information or posted at a public data repository, provide the repository URL if relevant, and provide specific details as to which raw blot/gel images, if any, are not available. Email us at plosone@plos.org if you have any questions.

The original gel images are downloaded as Supporting Information

3. We note that you have included the phrase “data not shown” in your manuscript. Unfortunately, this does not meet our data sharing requirements. PLOS does not permit references to inaccessible data. We require that authors provide all relevant data within the paper, Supporting Information files, or in an acceptable, public repository. Please add a citation to support this phrase or upload the data that corresponds with these findings to a stable repository (such as Figshare or Dryad) and provide and URLs, DOIs, or accession numbers that may be used to access these data. Or, if the data are not a core part of the research being presented in your study, we ask that you remove the phrase that refers to these data.

Phrase "data not shown" was removed from the new version of the manuscript

 4. Please include captions for your Supporting Information files at the end of your manuscript, and update any in-text citations to match accordingly. Please see our Supporting Information guidelines for more information: [http://journals.plos.org/plosone/s/supporting-information](about:blank).

Information about attached Supplementary files are placed at the end of our manuscript

5. Review Comments to the Author

Reviewer #1:

Thank you very much for the critical review of our manuscript. We agree with the majority (but not all) received comments. Looking at the comments, we understood that two serious problems with our work were pointed. First comment is connected with the way of investigation of protein secretion level, which was directly calculated from SEAP enzymatic activity. The second problem concerns the aim of this study. We tried to overcame those problems with newly introduced experiments and also by re-writing the text of the manuscript to be available for broader audience, for readers not only specialized in glycobiology. Below please find our detailed explanations for each comment expressed to the 1st version of our manuscript:

This article describes a modified alkaline phosphatase as secreted glycoprotein model. The alkaline phosphatase has been cloned into different mammalian expression plasmids and its expression/secretion into the medium was documented concerning N-Glycosylation driven by sites directed mutagenesis. Different expression systems, expression plasmids, and different purification columns were used.

Here are comments and major concerns about this article:

The title of this article is miss leading, this reviewer is concerned about the suitability of the title to the article contents “Modified secreted alkaline phosphatase - improved reporter protein for N-glycosylation analysis” how the secreted alkaline phosphatase do improve N-glycosylation analysis or vice versa?
We agree, the Title would be missleading. We changed it.

What were the hypothesis and aims of the current research?
Our work described genetic modification of plasmid (vector) for using in N-glycosylation analyses, the major type of protein post-translational modifications. The changed psiTEST plasmid was examined for its functionality in futher structural studies on N-glycans, sugar oligomers which are covalently attached to the recombined SEAP (secretion, occupancy of introduced sequons and improvements in purification from culture media were tested). We feel that researches not familiar with glycobiology are not aware with serious technical problems related to glycan analyses in various cells and tissues; it is well known that glycobiogists usually struggle to isolate representative pools of glycoconiugates. Glycosylation belongs to very common post-translational modifications, however, many important questions and problems, especially glycotransferase regulation, are still not known. Many strategies were proposed to overcame technical problems to possess reliable procedures for structural analysis of glycans, knowledge essential to investigate cellular glycosylation network. The choice of reporter, secretable glycoprotein in genetically modified cell line as a source of glycans was introduced many times in the past. In our oppinion, the manuscript presents one of the best system for such analyses, much better than previously proposed. In the Introduction chapter we add the new paragraph which explains all these problems. We hope this would help to understand the advantages of using such reporter glycoprotein for glycoanalyses.

The methods are not adequate for the specific results and conclusions!
The results are not in line with the conclusions of the article!

We hope that after introduced changes and additional experiments (see Figure 3), the conclusions, supplemented with additional comments match the results. From our point of view, now methods are also adequate for all presented results.

This reviewer doesn't see any relation between the glycosylation of alkaline phosphatase and its expression/activity as an enzyme unless the claims are tested one by one with proper methodology! If its expression, then the quantity of the enzyme expression and secretion in the medium should be tested with specific antibodies or other quantitation methods. If its activity, then, an equal amount of the enzyme and control one should be tested in the same assay.
We agree with the reviewer that the level of secretion must be analysed with more appropriate method. If the enzyme activity is used, all protein variants must express the same (or very similar) activity at standard conditions. We decided to analyse specific activity of three variants, examined in secretion experiments (see Materials and methods and Figure 3). Additionally the calculated molar absorbance coefficient, which was used to establish the concentration of homogenous SEAP for kinetic studies. Although we observed some, tiny differences between specific activities of SEAP variants, it is clear that this can not exclude enzyme activity measurments for estimation of the SEAP concentration in the culture medium.

What is the purpose of alkaline phosphatase as a reporter glycoprotein?
Alkaline phosphatase as a reporter enzyme how does this improve glycosylation analysis? The fact that alkaline phosphatase is a reporter enzyme and secreted into the cell culture medium makes it a single case in which its purification and analysis of its N-glycosylation are straightforward? So why is that so important?

We changed the Introduction section to explain why such methods are important, first of all as a real and representative source of mature N-glycans intended for future structural studies. We also proposed the optimal method of SEAP isolation, faster, efficient and ready for enzymatic de-glycosylation. The text of the manucript was supplemented with new paragraphs to be understable for broader audience.

The authors describe several expression systems such as HEK293, CHO, and HepJ cells indicating that these three expression systems are capable to express SEAP in different amounts! What is the purpose of this comparative data, how these data contribute to the article? And to the article aims if there are specific aims! How Figure 4 that depicts the N-glycosylation profile of the different expression system contributes to the article?

Here, we do not understand this comment. We feel that the reviewer did not know, that the three mentioned cell lines are frequently used in glycobiology. Especially CHO cell line, derived from Chinese Hamster ovary many years ago, looks as no.1 system for glycoanalyses, first of all because of avaliability of many glycomutants (for example Lec-type cell lines generated by dr. Pamela Stanley from late 80-ties last century to present), with disturbed glycosylation pathways. These cell lines were not accidentally choosen. Authors used to work with them for many years, and characterized them especially to analyse nucleotide sugar transporter action, the process which delivers substrates for glycosylation in ER and Golgi. But these profillings (now presented on figures 4 and 5) must be perfomed, for example to show, that the new genetic construct is not toxic for the tested cell lines and also to analyse the quality of isolated N-glycans, first of all, if the non-mature fraction is not dominating over mature N-glycans. Finally, our goal was not to compare the glycoprofiles but to show that the FULL procedure, from transfection to enzymatic de-glycosylation (see schematic figure 6) works well for all investigated lines. And even for the worse recorded secretion rate, it is clear that it may be apllied with success to get purified pool of N-glycans, detectable in chromatographic techniques after fluorescent labelling, ready for the next studies.

One of the authors' conclusions was “Additional N-glycosylation sites introduced by
site-directed mutagenesis significantly increased secretion of the protein”
Based on figure 2, this conclusion is incorrect! Figure 2 title is “Relative secretion level of SEAP from HEK293T cells transfected with modified psiTEST vector”. While on the Y-axis of figure 2 alkaline phosphatase activity is depicted, maybe additional glycosylation sites improved the activity but not the secretion of the enzyme, did the authors tested this claim? And how about the significance of the differences in activity between the site-directed mutagenesis and the wt enzyme? Is there any statistical analysis?

We add additional experiments to show similar phoshatase activity of all variants. Statistics was present in the previous version, now the manuscript is supplemented with additional information in the Materials and methods and also in the captions to figures 2 and 3.

Based on the data presented in figure 3, the authors concluded that the total cell lysate glycoproteins contain mannose immature glycoforms, but N-glycoforms of SEAP are mature types. Is that novel data? Is that surprising? How this specific work improves N-glycosylation analysis of secreted proteins while there is no additional information on the N-glycosylation types of SEAP?

Again, we fell that the reviewer is not familiar with glycobiology and glycotechniques. Yes, indeed, it is well known that the high level of high mannose type glycans is typical for many mammalian cell lines. For that reason researches are searching for other ways to analyse mature forms only (some examples of such strategies were explained in the Introduction). However, we undestand that readers of Plos One may be also puzzled and the objections from the reviewer may be admissible. Because of that, although there were some data about this phenomenon (domination of mannose structures) in the 1st version of the text, we decided to supplement the Introduction chapter to explain the necessity of looking for new, improved methods for isolation of mature glycans only.

Minor comments:
This article needs English editing
The introduction should be rewritten. The first two sentences of the introduction are saying the same!

We agree with the reviewer. The sentence was removed from the text. Introduction was rewritten to be more understable for general audience. We also made some minor language improvments in the text.

Introduction paragraph 4, “4) the reporter protein possesses at least three glycosylation sites. (the original SEAP contains two N-glycosylation sites, from which only one is occupied [15,16]), which usually makes N-glycan profile more complex”. What do you mean in this expressions? If only one glycosylation site is occupied does that make N-glycan profile more complex?

We agree with the comments, the sentence was modified.

Results and discussion:
The first paragraph “It seems that relatively big size of GST (25 kDa) would
be a negative factor for secretion of the enzyme.”
The authors should be careful by concluding that the reason for low secretion is that the protein size is big!

We agree, the sentence was removed. The reason of low activity/secretion of GST-tagged SEAP is not proved.

3.3 “In 6×His and HA constructs, we introduced 7 new N-glycosylation sites (14 constructs in total were prepared). First, all of them have been tested for secretion rate, using QUANTI-Blue reagent (Supplementary Table 1). The best plasmids were used as templates for the second round of site-directed mutagenesis to introduce additional N-glycosylation site (additional 12 double mutants in total were prepared).”
Rewrite please this is unclear, how many sites you have mutated in each construct?

For clarity, we transfer Table 1 from Supplementary material to the main text of the manuscript. In this table all tested constructs (including those from preliminary studies) are listed. We also made some changes in mentioned fragment of the manuscript.

3.3 the second paragraph “The effect of the same mutation in SEAP-HA construct was similar to 6×His plasmids or only slightly less efficient”
What do you mean by only slightly less efficient? Why it is important to state that?
These results of our preliminary experiments are summarised in Table 1, now the part of the main text. From our point of view the cited statement is important, because does not exclude HA tagged SEAP as a potential tool in future studies, for example to analyse secretion mashinery of selected cell lines, with the use of very specific, anti-HA antibodies, easily available on the market (this is impossible for 6xHis tag, due to low specificity of anti-6xHis antibodies). We struggled with purification of HA-tagged SEAP and stopped experiments on HA-SEAP as a source of glycans, however, we think that HA-SEAP still may be used in some experiments, if purification on immobilised HA-antibodies is avoided.

The last three lines in results and discussion “In contrast to high rate of glycans with terminally bound alphamannoses derived from the cell lysate glycoproteins of HEK293T (more than 80%), in SEAP only about 15% of total glycan content was detected as high-mannose type.
Where this data come from?

We included additional explanations in the Results an discussion part. In Materials and methods the phrase explaining the way of glycan pools calculations from fluorescent peak areas were added.

Reviewer #2: This is a well written manuscript describing a technique to measure glycosylation using secreted alkaline phosphatase modified to bear glycosylation sites inanition to the preexisting sites. Detailed methodology and conditions described will enable other researchers to use this technique. Overall it is an excellent paper. However I have the following comments.

Thank you very much for the critical review of our manuscript. We agree with the majority (but not all) received comments. Below please find the detailed anwers to the reviewer's concerns:

1. The legends for the figures are very brief making it difficult for someone who is not a glycobiologist to understand what the figures mean. Ex Fig 3 and 4. What do the axis mean and what is interpreted from the figure should be better described in the legend and better expanded in the text.

Description for Figures (now numbered as Figure 4 and 5) were changed to be more understable for general audience

2. One aspect that is confusing for this reviewer is whether glcolsylation sequence in the additional sites are the same as the glycosylation of the pre existing site. This could have been done with the protein modified to bear one additional site and compared to the preexisting site. I am assuming that the glycosylation sequence should be known for the preexisting sites. I am not sure what you gain from knowing glycosylation in all the new sites if we do not know if the new site glycosylation has no relation to glycosyl residue in the preexisting site.

We agree with the comment but this was not our goal to analyse all occupied N-glycosylation sites in details. The aim of this study was to improve the glycoprotein (SEAP, to be produced in at higher level, was easily purified in relatively short time, and be available for researches working in laboratories with standard equipment). We proved, that the final product contained 3 occupied glycosylation sites (see at higher molecular weight of constructs 278 and 150&278 visibleon figure 2 and figure 3) and was secreted into the medium very efficiently, more than initial SEAP.

3. It would have been interesting to choose to cell lines one from human and one from non human primate for comparison.

We agree with the reviewer, but our goal was not to analyse glycoprofiles of many cell lines. We use some cells to show functionality of the newly designed genetic vector. And we analysed two human cell lines and one, derived from Chinese Hamster ovary (CHO). These lines are typically used by researches for glycoanalyses. However, we are sure that the system may work for broad spectrum of mammalian cell lines, including non human primate. Assuming the high efficiency of transfection - we add some comments about that at the end od Results and discussion part.

4.What and how exactly the modified protein can be used could have been described in the discussion.

We agree that the previous version of the manuscript was very compact and might be not easy to understand by researches, not specialized in glycobiology. We added sugested paragraph at the end of Results and discussion section.
