# Supplementary material for: Modified secreted alkaline phosphatase as an improved reporter protein for N-glycosylation analysis
Source: PLoS One. 2021 May 25;16(5):e0251805. doi: 10.1371/journal.pone.0251805 (PMC8148361; doi:10.1371/journal.pone.0251805)
Supplement: Attachment — Submitted filename: Answers to reviewers comments, 2nd rev..docx [file pone.0251805.s006.docx]

Reviever 1

Thank you very much for your comments.

The manuscript was checked and improved by native English speaker.

Reviewer 2

No comments, the reviewer accepted the manuscript after 1st revision.
